# The Age-Specific Association of Waist Circumference and Risk of Chronic Kidney Disease in Patients with Type 2 Diabetes Mellitus in Shandong, China

**DOI:** 10.1155/2015/715871

**Published:** 2015-09-09

**Authors:** Lingling Xu, Weihong Yu, Ping Huang, Chunying Li, Yan Li, Meng Wang, Qun Xu, Jing Wang, Caixia Zheng, Bin Qu, Yanping Zhao, Meng Niu, Ou Wang, Fengying Gong

**Affiliations:** ^1^Department of Endocrinology, Key Laboratory of Endocrinology of the Ministry of Health, Peking Union Medical College Hospital, Chinese Academy of Medical Sciences, Beijing 100730, China; ^2^Department of Ophthalmology, Peking Union Medical College Hospital, Chinese Academy of Medical Sciences, Beijing 100730, China; ^3^Department of Ophthalmology, Traditional Chinese Medicine Hospital of Muping District of Yantai City, Yantai, Shandong 264100, China; ^4^Department of Endocrinology, Traditional Chinese Medicine Hospital of Muping District of Yantai City, Yantai, Shandong 264100, China; ^5^Department of Epidemiology and Biostatistics, Institute of Basic Medical Sciences, Chinese Academy of Medical Sciences and School of Basic Medicine, Peking Union Medical College, Beijing 100005, China

## Abstract

*Objective*. To examine the association of three most common obesity measures including body mass index (BMI), waist circumference (WC), and waist-to-hip ratio (WHR) with chronic kidney disease (CKD) risk in patients with type 2 diabetes mellitus (T2D). *Design*. Cross-sectional evaluation of the effect of anthropometric measures on CKD risk. *Setting*. Outpatient Department. *Subjects*. T2D patients who were treated between October 2012 and May 2013. *Intervention*. None. *Main Outcome Measure*. CKD risk. *Results*. On average, the patients had a mean age of 60.2 years, and 40% were males. CKD was present in 46% of all the patients. In multivariate logistic regression using the imputed data, higher WC was associated with greater odds of CKD (OR = 1.019, 95% CI = 1.002–1.006, *P* = 0.030), but not BMI and WHR. Interestingly, we found that patients with very small WC seemed to have greater odds of CKD. We observed age-specific effect of WC such that the effect of WC on CKD risk is significant only in middle-aged T2D patients. *Conclusion*. Our study provides evidence for the association of WC with CKD in Chinese patients with T2D. T2D patients, especially middle-aged T2D patients, should reduce their WC to decrease CKD risk.

## 1. Introduction

Chronic kidney disease (CKD) poses a significant public health challenge in China. It was recently reported that the overall prevalence of CKD was 10.8% in China [1]. Diabetes is one of the major risk factors for kidney damage, and people with diabetes have significantly increased risk for CKD. The prevalence of diabetes nephropathy in Asian type 2 diabetes mellitus patients was as high as 58.6% [2], and 63.9% of the Chinese patients diagnosed with type 2 diabetes (T2D) had CKD with varying stages [3].

Obesity is associated with both chronic kidney disease and T2D. It remains controversial whether common anthropometric measures of obesity, including body mass index (BMI), waist circumference (WC), and waist-to-hip ratio (WHR), are related to the risk of CKD. A recent research found U-shaped associations of these three obesity measures with urinary albumin-to-creatinine ratio (uACR) or microalbuminuria in the general population [4]. Another study showed that, among healthy men, higher baseline BMI was associated with increased risk for CKD [5]. In contrast, another research reported that WHR, but not BMI, was associated with incident CKD [6].

The relationship of these obesity measures to CKD is even less clear in patients with T2D, and findings in the literature are largely inconsistent and vary by populations. In the Look AHEAD (Action for Health in Diabetes) Study, increased BMI and WC were associated with albuminuria in overweight and obese adults with T2D [7]. However, an epidemiology study in UK showed that BMI was not related to the progression of CKD in patients with diabetes [8]. A study of 125 overweight T2D patients reported that waist-to-height ratio, rather than waist circumference, was associated with CKD [9]. Most of these studies were focusing on Caucasian participants. Compared to Caucasians, Asians have significantly higher percentage of body fat at lower mean BMI and are more likely to suffer obesity related diseases such as diabetes [10]. Prior studies on Asian patients showed that central obesity measures such as WC, but not BMI, were associated with 24-hour urinary albumin excretion rate and decline in renal function [11, 12]. To the best of our knowledge, there is only one study that investigated the association between anthropometric measures of obesity and CKD in patients with T2D in the Chinese population in Hong Kong [13]. In this study, we aimed to investigate the association of BMI, WC, and WHR with the risk of CKD in T2D patients in mainland China.

## 2. Materials and Methods

### 2.1. Subjects

This cross-sectional study included T2D patients who were treated in the Outpatient Department between October 2012 and May 2013. Patients were diagnosed with T2D if they (1) had no episodes of ketoacidosis; (2) were diagnosed with diabetes over 30 years of age; and (3) were not administrated with insulin during the first three years since the diagnosis of diabetes. For each patient, a personal interview was conducted to gather basic demographic data, including age, sex, education, smoking (never versus ever), and drinking status (never versus ever). Information about past medical history and time since the diagnosis of diabetes (months) was also collected.

The initial sample size consisted of 886 T2D mellitus patients who were 30 years of age and older. Four patients were excluded due to missing values for serum creatinine and/or urinary albumin, and an additional 12 patients had missing data on demographics or anthropometric measures. As a result, a total of 870 patients were included in this study. The study was conducted in accordance with the Declaration of Helsinki, and informed consent forms were obtained from all the patients.

### 2.2. Anthropometric Measurement of Obesity

Anthropometric measures included body height, weight, and waist and hip circumferences. Height was measured to the nearest 0.1 cm using a digital ultrasound instrument, and weight was measured to the nearest 0.1 kg in light clothing and without shoes using standard digital scales.

WC was measured to the nearest 0.1 cm between the lower rib margin and the iliac crest in the horizontal plane, with the subject standing comfortably with weight distributed evenly on both feet. Hip circumference was measured to the nearest 0.1 cm and was recorded as the greatest circumference around the gluteal region. Each measure was taken twice and the average was used for the analysis. BMI was calculated as the weight in kilograms divided by the square of height in meters. WHR was calculated as the ratio of WC divided by hip circumference. In this paper, we focus on the analysis of the three most common anthropometric measures, including BMI, WC, and WHR.

### 2.3. Diagnosis of CKD

Patients fasted for 8–12 hours overnight before their urine specimens and blood samples were collected in the morning. Blood samples were taken from the cubital vein of the patients in the supine position and prepared for immediate analysis or stored at −80°C for further analysis. Laboratory tests followed standard procedures. Briefly, plasma glucose was measured by glucose oxidase method. Plasma total cholesterol (TC) and triglyceride were measured by enzymatic methods. Hemoglobin A1c (HbA1c) was measured by high-performance liquid chromatography (HPLC) using the Bio-Rad D10 hemoglobin testing system. Urinary albumin was measured from fresh morning spot urine by the immunoturbidimetric assays and urinary creatinine was measured by the colorimetric method. Urinary albumin-to-creatinine ratio (uACR) was calculated as uACR = urinary albumin concentration (mg/L)/urinary creatinine concentration (mmol/L). uACR was measured twice and an average was taken. In cases when the two uACR measurements differed by more than 10%, another collection was made to assess the adequacy of the collections.

CKD was defined as estimated glomerular filtration rate (eGFR) < 60 mg/min/1.73 m^2^ or the presence of albuminuria. Here, eGFR was calculated using the Cockcroft-Gault equation: [(140-age (years)) × body weight (kg)/72 × serum creatinine (mg/dL)] × 0.85 (if female), and albuminuria was defined as uACR ≥ 2.7 mg/mmol.

### 2.4. Blood Pressure and Hypertension

Blood pressure (BP) was measured from the left arm with a mercury sphygmomanometer with the patient in a sitting position and after a 5-minute rest. BP was measured twice and an average was taken. Hypertension was defined as systolic blood pressure ≥ 140 mmHg or diastolic blood pressure ≥ 90 mmHg or the existence of a previous diagnosis of hypertension.

### 2.5. Statistical Analysis

We first compared demographic and clinical characteristics between patients with and without CKD. Chi-square test was used to compare the categorical variables, and continuous variables were compared using Student's *t*-test or Wilcoxon rank-sum test as appropriate. Multiple imputation was performed using the Amelia II package for variables with missing values [14]. We imputed missing data based on the other variables in the data and repeated the process 10 times, resulting in 10 imputed datasets.

The relation of anthropometric obesity measures to CKD was examined using multivariate logistic regression models with the presence of CKD as the binary outcome, adjusting for age, sex, education, smoking status (ever versus never), duration of diabetes, HbA1c, hypertension, and triglyceride. This was done using the 10 imputed datasets with the Zelig package [15, 16].

All data analyses were performed using R (http://www.R-project.org/) or SAS version 9.3 (SAS Institute Inc., Cary, NC) and we used a nominal threshold of *P* < 0.05 for statistical significance.

## 3. Results

A total of 870 patients were included in the analyses. The mean age was 60.2 ± 9.6  years (range: 30 to 88 years), and 40% were males. On average, they had 8.0 ± 3.1 years of education (range: 0 to 20 years). The mean time since the diagnosis of T2D was 9.9 ± 7.1 months (range: 0–91 months).

Of the 870 patients in the study, 402 (46.2%) had CKD. The comparison of the patients with and without CKD is presented in Table 1. Briefly, patients with CKD were, on average, older in age (*P* < 0.0001) and had fewer years of education (*P* = 0.001). Patients with CKD had higher WC and WHR (both *P*'s < 0.0001). Interestingly, we did not find a significant difference in BMI between patients with and without CKD (*P* = 0.493). Patients with CKD also had longer history of diabetes (*P* < 0.0001). Furthermore, compared to patients without CKD, patients with CKD were more likely to have hypertension (*P* < 0.0001); and they also had higher systolic and diastolic BP (both *P*'s < 0.0001). We found that patients with CKD had higher levels of blood glucose, both fasting and postprandial (both *P*'s < 0.0001), and higher HbA1c (*P* < 0.0001). Levels of serum triglyceride (*P* = 0.0003) and total cholesterol (*P* = 0.049) were also significantly higher among patients with CKD.

**Figure 1:**
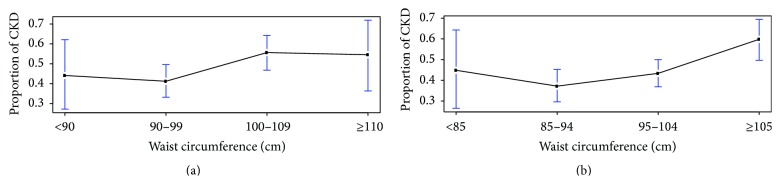
Proportion of chronic kidney disease among type 2 diabetes patients with different waist circumferences. (a) Proportion of CKD among male T2D patients. (b) Proportion of CKD among female T2D patients. CKD: chronic kidney disease; T2D: type 2 diabetes; WC: waist circumference.

**Figure 2:**
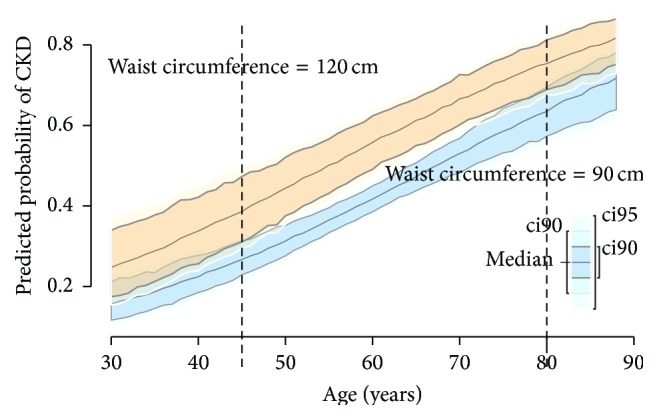
Effect of waist circumference on chronic kidney disease risk by age. We compared the CKD risk for those having WC = 120 cm with those having WC = 90 cm. The *x*-axis represents the age of the T2D patients, and the *y*-axis represents the estimated probability of CKD risk. The solid lines represent the estimated probabilities, the dashed line corresponds to age = 45 and age = 80, respectively, and the shaded areas represent the corresponding confidence intervals. WC: waist circumference; CKD: chronic kidney disease; T2D: type 2 diabetes.

We conducted logistic regression analysis to examine the association of BMI, WC, and WHR with CKD (Table 2), adjusting for age, sex, education, smoking status (ever versus never), time since diagnosis of diabetes, HbA1c, hypertension, and triglyceride. We did not find a significant association of BMI with CKD (OR 1.010, 95% CI: 0.978–1.043, *P* = 0.550). Similarly, WHR was not associated with CKD (OR 1.191, 95% CI: 0.926–1.532, *P* = 0.174). On the other hand, higher WC was associated with greater odds for CKD such that, for every 1 cm increase in the circumference, the odds of having CKD increased by approximately 2% (OR = 1.019, 95% CI = 1.002–1.036, *P* = 0.030). The relationship between WC and CKD is further illustrated in Figure 1. Surprisingly, patients with small WC (<90 cm in males and <85 cm in females) had a relatively high proportion of CKD. In males, the proportion of CKD reached a plateau for those with WC ≥ 100 cm, while in females, among those with WC ≥ 85 cm, the proportion of CDK increases as WC increases. We performed sensitivity analysis by excluding male subjects with WC < 90 cm and female subjects with WC < 85 cm and found similar association between WC and CKD (OR 1.028, 95% CI 1.006–1.051, *P* = 0.012). A further examination by age indicates that the effect of WC on CKD risk is most significant for middle-aged patients, while, for patients aged 45 years or younger and those aged 80 years or older, there is no dramatic difference in CKD risk between those having large WC and those having small WC (Figure 2).

## 4. Discussion

In this cross-sectional study, using data from 870 patients with T2D, we examined the association of obesity measures with the risk of CKD. We found that, as a central obesity measure, WC was associated with greater odds for CKD, independent of known CKD risk factors, and the association is age specific such that the effect of WC on CKD risk is most significant for middle-aged patients. We did not find significant association of BMI and WHR with risk of CKD. Our results contribute to the research on CKD among patients with T2D in several important ways.

Our findings lend empirical support that, in Chinese patients with T2D, central obesity measure WC is more strongly associated with CKD than BMI. Previous literatures on whether BMI increases the risk of CKD remain controversial. Data from a clinical trial in the US supports that both BMI and waist circumference are associated with increased level of albuminuria [7]. In contrast, a study of 1760 outpatients with T2D in Italy found that BMI does not influence the progression rate of CKD in patients with T2D [17]. A separate study in the US reported a similar finding where WHR, but not BMI, is associated with incident CKD [18]. Another study reported that higher BMI reduced the deterioration of renal function in T2D patients with CKD stage 3 or 4 by reducing glomerular filtration rate decline [19].

One possible reason for the inconsistency in the findings can be attributable to the “obesity paradox” where BMI, as a measure of overall level of adiposity or general obesity, does not distinguish fat and muscle mass, while more muscle mass results in higher serum creatinine concentration [20]. On the other hand, central obesity measures, such as WC and WHR, serve as better markers for visceral fat in CKD [21]. Separately, the difference in the patient populations may also contribute to the disparity of the findings. Compared with the white population, Asian population in general had a higher percentage of body fat at a lower BMI [22, 23]. A recent study reported that, in Korea, central obesity measures were associated with decline in renal function, but not BMI [12]. However, data from the Hong Kong Diabetes Registry showed that higher BMI was protective against the risk of incident CKD in Chinese patients with T2D [13]. Another study in Turkey examined the relationship of four anthropometric measures (BMI, WC, WHR, and conicity index) with 24-hour urinary albumin excretion rate and found that only WC exhibited independent association [11]. A recent study in Iran found that, in men, waist gain but not decrease in WC was associated with CKD risk, but no association of WC with CKD risk was found in women [24]. These studies imply racial/ethnic disparities in the association of various obesity measures with risk of CKD.

Interestingly, we found higher proportion of CKD in patients with very low (<90 cm in men or <85 cm in women) or very high WC (≥110 cm in men or ≥105 cm in women; Figure 2). This is consistent with findings from a recent study which reported higher odds of CKD in patients with low or high quintiles of WC [4]. The exact mechanism underlying this phenomenon is unclear. Previous studies found a U-shape between BMI and all-cause mortality such that low or high BMI is associated with high mortality risk while the lowest mortality risk is reached for BMI between 22.5 and 25 kg/m^2^ [25]. Low anthropometry might represent an alteration of internal hemodynamics and patients with low WC may suffer damaged glomerulus and have different pathophysiology compared to obese patients [4, 25, 26].

There are many methodological concerns underlying the inconsistency such as definition of eGFR [27]. Previous researches imply that part of the reasons accounting for the discrepancy between the results regarding the relationship between central obesity and CKD risk might be due to different age distribution of the included participants. For example, a previous study in China that found WC was associated with increased CKD risk included subjects aged 35–74 years [28], while two other studies in China that failed to find significant association included younger subjects (≥30 years old [29] and ≥20 years old [27]). Few studies have examined the age-specific association of central obesity with CKD risk, and our results indeed indicate that the effect of WC varies by age. We speculate that the insignificant effect of WC on CKD risk in young and elder patients might be due to the fact that (1) young patients had relatively short duration of diabetes, which was a significant risk factor of CKD (*P* = 0.01), leading to less likelihood of developing CKD, and (2) older patients might suffer muscle loss and, as a result, the commonly used obesity measures, such as WC, might not be applicable to them [30]. More longitudinal studies are needed to validate the age-specific effect of WC on CKD risk.

Our study has limitations. First, although we have controlled potential confounders, the possibility of residual confounding by other unknown or unmeasured factors cannot be ruled out. Second, the cross-sectional nature of this study precludes analysis of longitudinal data and any causal inference. Finally, participants of this study came from a single hospital, and our findings may not be generalizable to other populations.

## 5. Conclusions

In summary, in this study, we examined the association of obesity measures with CKD risk in patients with T2D. We found that WC is significantly associated with CKD, independent of known CKD risk factors, but no association was found for BMI, indicating that central obesity, but not general obesity, might contribute to the pathogenesis of CKD. Future studies are needed to validate our findings and to explore the mechanism underlying the observed association.

## Figures and Tables

**Table 1:** Characteristics of the study patients.

	Overall (*N* = 870)	CKD^−^ (*N* = 468)	CKD^+^ (*n* = 402)	*P*
Age (yr)	60.3 (9.6)	57.9 (8.7)	62.9 (9.8)	<0.0001
Male sex	348 (40.0%)	180 (38.5%)	168 (41.8%)	0.318
Education (yr)	7.9 (3.2)	8.2 (3.0)	7.5 (3.3)	0.001
Smoker (%)	130 (15.0%)	68 (14.6%)	62 (15.5%)	0.711
Height (cm)	163.6 (7.8)	164.0 (7.7)	163.2 (7.9)	0.113
Weight (kg)	73.3 (14.1)	73.4 (14.4)	73.2 (13.8)	0.877
BMI (kg/m^2^)	27.3 (4.6)	27.2 (4.8)	27.4 (4.5)	0.493
WC (cm)	98.1 (9.1)	96.9 (8.7)	99.4 (9.4)	<0.0001
WHR	0.96 (0.06)	0.96 (0.06)	0.97 (0.06)	<0.0001
Time since diagnosis of T2D (months)	9.9 (7.1)	8.6 (6.8)	11.4 (7.2)	<0.0001
Hypertension	489 (56.2%)	229 (48.9%)	260 (64.7%)	<0.0001
Systolic blood pressure (mmHg)	136.8 (15.6)	133.8 (14.2)	140.2 (16.4)	<0.0001
Diastolic blood pressure (mmHg)	83.6 (8.9)	82.9 (8.2)	84.5 (9.5)	0.006
FBG (mmol/L)	9.6 (4.4)	9.0 (3.0)	10.3 (5.5)	<0.0001
PBG (mmol/L)	13.0 (4.1)	12.4 (3.9)	13.7 (4.2)	<0.0001
Triglyceride (mmol/L)	1.46 (1.55)	1.39 (1.81)	1.55 (1.18)	0.0003
Total cholesterol (mmol/L)	5.7 (2.6)	5.5 (1.3)	5.8 (3.6)	0.049
Hemoglobin A1c (%)	8.1 (1.9)	7.8 (1.7)	8.4 (2.0)	<0.0001

Data were presented as mean (SD) or *N* (%).

SD: standard deviation; CKD^−^: T2D patients without CKD; CKD^+^: T2D patients with CKD; CKD: chronic kidney disease; BMI: body mass index; WC: waist circumference; WHR: waist-to-hip ratio; T2D: type 2 diabetes; FBG: fasting blood glucose; PBG: postprandial blood glucose.

**Table 2:** Associations of anthropometric measures with chronic kidney disease in patients with type 2 diabetes.

Variable	OR (95% CI)	*P* value
BMI	1.010 (0.978–1.043)	0.550
WC	1.019 (1.002–1.036)	**0.030**
WHR	1.191 (0.926–1.532)	0.174

Results were obtained in separate logistic regressions using 10 imputed datasets, adjusting for age, sex, education, smoking status (ever versus never), duration of diabetes, HbA1c, hypertension, and triglyceride.

*P* values in bold font indicate statistical significance.

OR: odds ratio; CI: confidence interval; BMI: body mass index; WC: waist circumference; WHR: waist-to-hip ratio.
